# Prevalence of Thinness and Stunting and Associated Factors among Adolescent School Girls in Adwa Town, North Ethiopia

**DOI:** 10.1155/2016/8323982

**Published:** 2016-05-16

**Authors:** Tsgehana Gebregyorgis, Takele Tadesse, Azeb Atenafu

**Affiliations:** ^1^Department of Human Nutrition, Institute of Public Health, College of Medicine and Health Sciences, University of Gondar, P.O. Box 196, Gondar, Ethiopia; ^2^Department of Environmental and Occupational Health, Institute of Public Health, College of Medicine and Health Sciences, University of Gondar, P.O. Box 196, Gondar, Ethiopia

## Abstract

*Introduction*. Despite the fact that adolescence is a window of opportunity to break the intergenerational cycle of malnutrition, adolescents are the neglected age groups. Hence information regarding the nutritional status of adolescents is lacking making creating and implementing intervention programs difficult.* Objective*. To assess the prevalence of thinness, stunting, and associated factors among adolescent school girls in Adwa town, Northern Ethiopia.* Methods*. Data on 814 adolescent female students were collected from March to April 2015 using interviewer administered pretested semistructured questionnaire and anthropometric measurements. Data were entered using EPI INFO version 3.5.3 and analyzed using SPSS version 20 and WHO Anthroplus software.* Results*. The prevalence of thinness and stunting was 21.4% and 12.2%, respectively. Age of adolescent [AOR = 2.15 (1.14,4.03)], mother's educational status [AOR = 2.34 (1.14,4.80)], eating less than 3 meals per day [AOR = 1.66 (1.12,2.46)], having family size >5 [AOR = 2.53 (1.66,3.86)] were significantly associated with thinness among the adolescent girls. Family size >5 [AOR = 2.05 (1.31,3.23)] and unimproved source of drinking water [AOR = 3.82 (2.20,6.62)] were significantly associated with stunting.* Conclusion and Recommendation*. Thinness and stunting are prevalent problems in the study area. Strategies to improve the nutritional status of girls should be given much attention.

## 1. Introduction

Adolescents constitute 20% of the world population and are estimated to be 1.13 billion by the year 2025 [1]. About 25% of the Ethiopian population are adolescents [2].

Adolescence is a period of rapid growth and development by which up to 45% of skeletal growth takes place and 15 to 25% of adult height is achieved [1, 3]. In addition to the increased nutritional requirements during adolescence period, poor dietary diversity and dietary inadequacies are more likely threats among adolescents due to their erratic eating pattern and having specific psychosocial factors [1, 4, 5].

Malnutrition passes from generation to generation, because adolescent girls that enter pregnancy with poor nutrient store are more likely to give birth to low birth weight or intrauterine growth restricted baby that is more vulnerable to metabolic disorders later in life [6]. So adolescence period is a unique opportunity to break a range of vicious cycles of structural problems that are passed from one generation to the next, such as poverty, gender discrimination, violence, poor health, and nutrition [1, 4].

Study done in Khagrachari District, Bangladesh, shows that 13.67% of adolescent girls were severely stunted and 20.33% were moderately stunted [7]. Another study done in rural areas of Bangladesh shows that 32% of the adolescent girls were stunted [8].

According to different studies done in India the prevalence of stunting ranges from 11.7% to 34.2% [9–12].

Studies done in Asembo and Mumias, Kenya, and Tunisia reported that the prevalence of thinness was 15.6% and 1.3%, respectively [13, 14]. According to the study done on the rural communities of Tigray, Ethiopia, 58.3% of the adolescent girls were thin [15].

Age, family size, mother education status, wealth index, lack of latrine were the commonly mentioned factors that influence the nutritional status of the adolescents. Despite the fact that adolescents are future mothers and need critical attention, they are the most neglected age groups [1, 4]. The intergenerational cycle of malnutrition has to be broken by strategies to improve nutrition of adolescents. There is limited information about the nutritional status and associated factors in adolescent girls in Ethiopia especially including pubertal landmarks which is critical for creating strategies and interventions on these target groups. Therefore, this study will address the gap by assessing the nutritional status and associated factors of adolescent school girls in Adwa town.

## 2. Methods

This study was conducted from March to April 2015 to assess prevalence of thinness, stunting, and associated factors among adolescent school girls in Adwa town, Ethiopia. Adwa town is in Central Tigray zone, Northern Ethiopia, which is 935 Km from Addis Ababa, Ethiopia. In the academic year 2014/2015 the total number of adolescent girls from grade 4 to grade 12 was 5974 of which 5603 were from government schools and the remaining 371 are from nongovernment schools (Adwa town education office). The study utilized institution-based cross-sectional study design with quantitative data collection method and anthropometric measurements. The study population included all regular adolescent girls attending grades 4–12 who were randomly selected by computer generated method in the randomly selected schools. Adolescent school girls who were seriously ill and unable to stand by themselves were excluded from the study.

The sample size was calculated using single population proportion formula with the assumptions of prevalence of thinness 58.3%, which was obtained from research conducted among rural adolescent girls of rural community of Tigray, Ethiopia [15]. Using 5% margin of error, 95% confidence level, 10% nonresponse rate, and the design effect of two the calculated sample size was 823, but due to the 1.09% nonresponse rate information was collected from 814 students, by which 732 were from government schools and 82 from private schools.

The sample was obtained using multistage sampling technique. During the first stage, schools were stratified into government and private schools. In the second stage from government schools 2 primary schools, 1 high school, and one preparatory school were selected by using lottery method out of 10 primary schools, 3 high schools, 2 preparatory schools, and 1 private school, which was included purposively since it was the only school from grade 4 to grade 12.

Finally, the total sample size was distributed proportionally to the selected schools based on the total number of students in each school. Individual students from each school were selected by using simple random sampling technique using computer generated random numbers. The sampling frame was students' identification number in their respective school.

The questionnaire was pretested on 5% (41) adolescent girls in a school other than those included in the sample. Data collectors and supervisors were trained for one day to have consensus and same understanding of what is intended to be measured by each question in the questionnaire. All the interviewers and supervisors were able to communicate in the local language, Tigrigna.

The study was reviewed and approved by Institution Research Review Boards, Institute of Public Health at the University of Gondar. Individual assent was obtained by explaining the purpose and the importance of the study to the adolescent girls. Parental consent from the parents was obtained by sending consent form with the students the day prior to the data collection. Moreover, confidentiality of the information was assured by using anonymous questionnaires and by keeping the data in a secured place.

Data were collected by interviewing the respondents through semistructured questionnaire and by anthropometric measurements. Interviewer administered questionnaire was used to collect information on the sociodemographic and socioeconomic status. Family income of the adolescent girls was assessed by using wealth index which is a good method of assessing economic status of a family.

Stadiometers with a sliding headpiece attached to digital weight scale were used to measure height and weight, respectively. Height was measured to the nearest 0.1 cm and weight to the nearest 0.1 kg in standing position. Each subject was weighed with minimum clothing and no foot wear. The scales were carefully handled and periodically calibrated by placing standard calibration weights of 2 kg iron bars on the scale. If the scale weight did not match the calibration weight, the scale was calibrated by adjusting its calibration screw while the calibration weight was on the scale. To avoid variability among the data collectors, the same specifically trained measurers were employed for anthropometric measurement. Anthropometric measurements were converted to height-for-age and BMI-for-age *Z* scores by using Antro Plus software. Girls with height-for-age below −2*Z* scores and BMI-for-age below −2*Z* scores of the 2007 WHO reference population were classified as stunted and thin, respectively [16].

### 2.1. Definition of Terms

The definitions of terms are as follows:

 thinness: BMI-for-age <−2*Z* scores of the 2007 WHO reference;
 stunting: height-for-age < −2*Z* scores of the 2007 WHO reference;
 adolescents: individuals in the age group of 10–19 years of age:
   early adolescents: adolescents in the age group of 10–13 years of age;
   middle adolescents: adolescents in the age group of 14–16 years of age;
   late adolescents: adolescents in the age group of 17–19 years of age;
 poor dietary diversity: adolescent girls with dietary diversity score below the median value (<4 food groups);
 good dietary diversity: adolescent girls with dietary diversity score of the median and above the median values (≥4 food groups);
 high mass media exposure: adolescent girls who had listened to radio or watched television or read newspaper/magazines at least once a week;
 low mass media exposure: adolescent girls who had not listened to radio or watched television or read newspaper/magazines at least once a week;
 improved source of water: including tap water, public tap, and protected well;
 nonimproved source of water: including unprotected spring and unprotected well.

The dependent variables were thinness and stunting while the following factors were included in the model as independent variables: sociodemographic characteristics (age, wealth index, family size, mother education, father education, place of residence, and type of school), menstruation status, physical activity, meal pattern, dietary diversity, source of drinking water, availability of home latrine, and mass media exposure.

Bivariate analysis was done and variables with *p* value less than 0.2 were included in the multiple logistic regression analysis. Odds ratio and 95% confidence intervals were also computed along with the corresponding *p* value.

## 3. Results

From the total of 823 adolescent girls, 814 responded to the questionnaire making the response rate 98.9%. The mean age of the study participants was 15.27 years (15.27 ± 2.05 SD). Seven hundred thirty-two (89.9%) of the respondents were from government schools and the remaining 82 (10.1%) were from private schools. Three hundred thirty-three (40.9%) of the mothers of the study subjects had no formal education followed by secondary level of education, 181 (22.3%). Nearly one-quarter, 210 (25.8%), of respondents were found in the first quintile range of wealth index (Table 1).

### 3.1. Dietary, Medical, Lifestyle, and Reproductive Factors

From the total of 814 respondents, 543 (66.7%) of them usually ate 3 or more meals per day. Four hundred twenty-three (52.0%) of the respondents had good dietary diversity and the remaining 391 (48.0%) had poor dietary diversity score.

From the total respondents, 97 (11.9%) had history of illness in the past two weeks prior to the data collection. Regarding physical activity of the respondents, 800 (98.3%) and 561 (68.9%) were involved continuously in walking for >30 minute per day and moderate intensity sport activities for ≥10 minutes continuously per day, respectively. Six hundred three (74.1%) of the respondents start their menstruation.

### 3.2. Household Environmental Factors

Seven hundred fifteen (87.8%) of the respondents used drinking water from improved source. Four hundred fifty (55.3%) and 661 (81.2%) of the study subjects reported that home gardening and home latrine were available in their home, respectively.

### 3.3. Nutrition Related Factors

Of the total 814 respondents, 671 (82.4%) had high mass media exposure and the remaining 143 (17.6%) had low mass media exposure. Five hundred sixty-five (69.4%) of the respondents had information about adolescent nutrition and 249 (30.6%) did not have information about adolescent nutrition.

### 3.4. Prevalence of Thinness and Stunting

**Figure 1:**
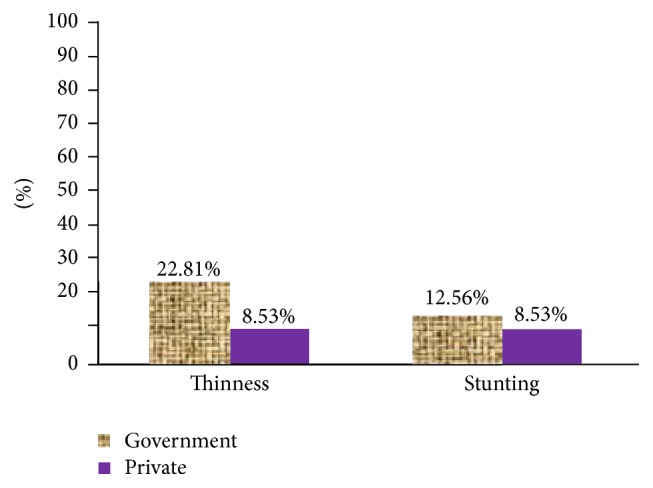
Prevalence of thinness and stunting among governmental and private adolescent school girls of Adwa town, Northern Ethiopia, 2015.

The overall prevalence of thinness and stunting among adolescent school girls of Adwa town was 21.4% (95% CI = 18.5%, 24.2%) and 12.2% (95% CI = 9.9%, 14.4%), respectively. The school specific prevalence of thinness and stunting is shown in Figure 1.

### 3.5. Factors Associated with Thinness

The odds of thinness were 2.15 times higher among adolescent girls in the early adolescent stage as compared to adolescent girls in the late adolescent stage [AOR (95% CI) = 2.15 (1.14,4.03)].

The odds of thinness were higher among adolescent girls enrolled in government schools. Adolescent girls in government schools were 2.89 times more likely to be thin as compared to adolescent girls from private schools [AOR (95% CI) = 2.89 (1.20,6.91)].

The odds of thinness were 3.27 [AOR (95% CI) = 3.27 (1.98,5.40)] times higher among adolescent girls who use water from unimproved source as compared to adolescent girls who use water from improved source (Table 2).

### 3.6. Factors Associated with Stunting

Adolescent girls who were from family size of greater than five were 2.05 times more likely to be stunted as compared to adolescent girls from family size of less than or equal to five [AOR (95% CI) = 2.05 (1.31,3.23)].

Adolescent girls who did not start menstruation were 2.80 [AOR (95% CI) = 2.80 (1.75,4.48)] times more likely to be stunted as compared to adolescent girls who started menstruation (Table 3).

## 4. Discussion

The prevalence of thinness among the study participants was 21.4%. This prevalence is higher when compared to studies done in Asembo and Mumias, Kenya (15.6%), Tunisia (1.3%), and 10% of Tamale Metropolis, Ghana [13, 14, 17]. The possible explanation for this difference could be due to difference in the study group since unlike this study which assessed the early, middle, and late stages of adolescent, study done in Tunisia considered adolescents in the middle and late stages which are less likely to be thin because of less possibility of height growth than early adolescents. The variation could also be due to socioeconomic and cultural difference in dietary habit and care practices.

The prevalence in this study is lower than the findings of studies done in Bangladesh, Tamil Nadu and west Bengal, India, and Tigray, Ethiopia, where 26%, 28.2, 40.94%, and 58.3% of the adolescent girls were thin, respectively [8, 10, 12, 15]. This difference might be due to urban rural difference since, unlike this study which included adolescent school girls from both urban and rural setting, the above studies included adolescent girls only from rural settings which are more likely to be involved in activities which need more energy expenditure.

The prevalence of stunting among the study participants was 12.2%. This finding is lower than the findings of studies done in West Bengal and Tamil Nadu, India, urban area of Umuahia, Nigeria, and Tigray, Ethiopia, by which 34.2%, 19.2%, 57.8%, and 26.5% of the adolescents were stunted, respectively [10, 12, 15, 18]. This difference might be due to the difference in the study setting by which, unlike this study which included urban and rural adolescent girls, the above studies were done in rural areas by which unhealthy household environmental factors are more likely to be common than the urban setting. The difference could also be due to time gap variation as nowadays knowledge of parents under nutrition and its consequence is improving.

The likelihood of being thin was higher among adolescents in the early and middle stage of adolescence than late stage of adolescence. Adolescent girls in the early stage of adolescence were 2.15 times more likely to be thin as compared to adolescent girls in the late stage of adolescence. This finding is similar with findings from Belgaum and Karnataka, India, and Tigray, Ethiopia [15, 19]. This might be due to the increased growth spurt during the early and middle adolescent stage compared to late adolescent with sudden increase of height in the early and middle adolescents than late adolescents.

Type of school was significantly associated with thinness in this study. Adolescent girls enrolled in government school were 2.89 times more likely to be thin as compared to adolescent girls from private school. This finding is in line with findings from Delhi, India, and Jimma, Ethiopia [20, 21]. This might be due to differing school environment by which government schools environment is less hygienic than the private school predisposing students to infection that leads to poor nutritional status. Other reasons could be that government schools include students who came from rural areas involved in higher energy expenditure activities but private school students are mainly from urban areas. This finding also could be due to poor economic status of government school adolescent girls' parents.

Adolescent girls who usually eat less than 3 meals per day are 1.66 times more likely to be thin as compared to adolescent girls who usually eat 3 or more meals per day. This finding is in line with the finding from Islamabad city, Pakistan, and Tamale Metropolis, Ghana [17, 22]. This might be due to skipping of meals leading to inadequate dietary intake. Adolescence period has the fastest growth and the nutritional requirements are increased to promote this growth. So, in addition to the increased nutritional demand during adolescent period, skipping of meals leads to being thin.

Adolescent girls who were from family size of greater than five were about 2.53 times more likely to be thin as compared to adolescent girls from family size of less than or equal to five. This finding is in line with findings from Tamil Nadu, Delhi, and Assam, India, and Jimma, Ethiopia [10, 11, 20, 21]. This might be due to having more family members which could lead to sharing of the available food for the large household members causing inadequate consumption of food leading to being thin. This finding also could be due to increased family size mostly occurring in uneducated parents who are more likely to accept and practice food taboos affecting mostly females.

Adolescent girls whose mothers did not have formal education were 2.34 times more likely to be thin as compared to those whose mothers have completed college and higher education. This is in line with study done in Assam, India [11]. This is due to the fact that if the level of education of the mother is low, her decision making and her contribution to the total family income will be low. This places the family at risk of not meeting their needs including nutritional needs.

Adolescents who use drinking water from unimproved source are 3.27 times more likely to be thin as compared to adolescent girls who use drinking water from improved source. This might be due to the fact that impure water is vehicle for intestinal parasites which leads to loss of appetite leading to poor nutritional status.

Family size was significantly associated with the risk of being stunted. Adolescent girls who were from family of greater than five individuals were 2.05 times more likely to be stunted as compared to adolescent girls from family of less than or equal to five individuals. This finding is in line with the findings from Tamil Nadu and Assam, India, and Jimma, Ethiopia [10, 11, 21]. This might be due to sharing of the available food for the large household members causing inadequate consumption of food leading to be stunted. Adolescent girls who did not start menstruation were 2.80 times more likely to be stunted as compared to adolescent girls who started menstruation. This is in line with the findings of studies done in Mumias and Asembo, Kenya, and Wannune Benue state, Nigeria [13, 23]. This might be explained by the fact that starting menstruation coincides with the adolescent growth spurt. Delay in menstruation in stunted adolescents shows the opportunity for catch-up growth as stunting delay menarche.

Another factor significantly associated with being stunted was source of drinking water. The odds of stunting were 3.82 times higher among adolescent girls who use water from unimproved source as compared to adolescent girls who use water from improved source. This might be due to repeated infection causing depressed immunity and making the severity and duration of disease more sever contributing to poor nutritional status of the adolescents.

## 5. Conclusion

Thinness and stunting are prevalent problems in the study area. Age of the respondent, type of school enrolled, meals eaten per day, education status of mother, source of drinking water, and family size were significantly associated with thinness among the respondents. Menstruation status, family size, and source of drinking water were significantly associated with stunting among respondents. The result of this study helps in understanding of the magnitude of the problem in the area. Programs should be implemented to decrease this problem at all level. As results from different study show micronutrient supplementation for adolescent girls should be provided to improve their nutritional status.

A major strength of this study was the random selection of the schools and adolescents. Generalization may be made to adolescent girls in the study areas as an attempt was made to identify randomized schools and adolescents from the study area. Another strength of this study was the use of dietary diversity as a proxy measure of micronutrient adequacy. The major limitation of this study was the failure to collect information on variables like food taboos and carrying food to schools. Another limitation of the study was the use of cross-sectional study design which makes any inference of growth pattern over time difficult. The cross-sectional nature of the study could only generate a hypothesis about the possible role of certain independent variables on the nutritional status of these adolescent girls but not their causal relationships.

## Figures and Tables

**Table 1:** Sociodemographic characteristics of adolescent school girls, Adwa town, Northern Ethiopia, 2015.

Characteristics	Frequency	Percentage (%)
Age		
Early adolescents	142	17.4
Middle adolescents	453	55.7
Late adolescent	219	26.9
Religion		
Orthodox	758	93.1
Muslim	52	6.4
Catholic	4	0.5
Place of residence		
Urban	512	62.9
Rural	302	37.1
Father's education		
No formal education	220	27.0
Primary education	213	26.2
Secondary education	205	25.2
College and above	176	21.6
Father's occupation		
Government employee	248	30.5
Farmer	315	38.7
Daily labourer	42	5.2
Merchant	199	24.4
Other^*∗*^	10	1.2
Mother's education		
No formal education	333	40.9
Primary education	168	20.6
Secondary education	181	22.3
College and above	132	16.2
Family size		
≤5	446	54.8
>5	368	45.2
Wealth index		
1st quintile	210	25.8
2nd quintile	200	24.6
3rd quintile	184	22.6
4th quintile	220	27.0

*∗* = NGO workers and private workers.

**Table 2:** Bivariate and multivariate logistic regression of factors associated with thinness among adolescent school girls, Adwa town, Northern Ethiopia, 2015.

Characteristics	Thinness		COR (95% CI)	AOR (95% CI)
	Yes	No		
Age of adolescent				
Early	33 (23.2%)	109 (76.8%)	1.98 (1.14, 3.44)	2.15 (1.14, 4.03)^*∗*^
Middle	112 (24.7%)	341 (75.3%)	2.15 (1.37, 3.35)	1.96 (1.20, 3.18)^*∗*^
Late	29 (13.2%)	190 (86.8%)	1	1
Place of residence				
Urban	84 (16.4%)	428 (83.6%)	1	
Rural	90 (29.8%)	212 (70.2%)	2.16 (1.54, 3.03)	1.31 (0.59, 2.93)
Type of school				
Governmental	167 (22.8%)	565 (77.2%)	3.16 (1.43, 7.00)	2.89 (1.20, 6.91)^*∗*^
Private	7 (8.5%)	75 (91.5%)	1	1
Education status of mother				
No formal	96 (28.8%)	237 (71.2%)	3.7 (1.99, 6.89)	2.34 (1.14, 4.80)^*∗*^
Primary	37 (22.0%)	131 (78.0%)	2.58 (1.31, 5.09)	1.78 (0.86, 3.66)
Secondary	28 (15.5%)	153 (84.5%)	1.67 (0.83, 3.374)	1.29 (0.61, 2.73)
College and above	13 (9.8%)	119 (90.2%)	1	1
Meals per day				
<3 meals/day	80 (29.5%)	191 (70.5%)	2.00 (1.42, 2.81)	1.66 (1.12, 2.46)^*∗*^
≥3 meals/day	94 (17.3%)	449 (82.7%)	1	1
History of illness				
Yes	31 (32.0%)	66 (68.0%)	1.88 (1.18, 3.00)	1.40 (0.83, 2.35)
No	143 (19.9%)	574 (80.1%)	1	1
Home latrine				
Yes	111 (16.8%)	550 (83.2%)	1	1
No	63 (41.2%)	90 (58.8%)	3.46 (2.36, 5.07)	1.54 (0.91, 2.59)
Family size				
≤5	59 (13.2%)	387 (86.8%)	1	1
>5	115 (31.2%)	253 (68.8%)	2.98 (2.09, 4.23)	2.53 (1.66, 3.86)^*∗*^
Source of drinking water				
Improved	126 (17.6%)	589 (82.4%)	1	1
Nonimproved	48 (48.5%)	51 (51.5%)	4.4 (2.83, 6.82)	3.27 (1.98, 5.40)^*∗*^
Wealth quintile				
1 quintile	41 (19.5%)	169 (80.5%)	1.02 (0.63, 1.66)	0.95 (0.54, 1.70)
2 quintile	55 (27.5%)	145 (72.5%)	1.60 (1.01, 2.54)	1.15 (0.66, 1.99)
3 quintile	36 (19.6%)	148 (80.4%)	1.03 (0.62, 1.69)	0.95 (0.54, 1.66)
4 quintile	42 (19.1%)	178 (80.9%)	1	1

*∗* = *p* value < 0.05.

**Table 3:** Bivariate and multivariate logistic regression of factors associated with stunting among adolescent school girls, Adwa town, Northern Ethiopia, 2015.

Characteristics	Stunting			
	COR (95% CI)		AOR (95% CI)	
	Yes	No	Yes	No
Age of adolescent				
Early	28 (19.7%)	114 (80.3%)	2.91 (1.53, 5.56)	1.62 (0.64, 4.09)
Middle	54 (11.9%)	399 (88.1%)	1.60 (0.90, 2.84)	1.25 (0.68, 2.30)
Late	17 (7.8%)	202 (92.2%)	1	
Type of school				
Governmental	92 (12.6%)	640 (87.4%)	1.54 (0.68, 3.44)	
Private	7 (8.5%)	75 (91.5%)	1	
Meals per day				
<3 meals/day	45 (16.6%)	226 (83.4%)	1.8 (1.17, 2.76)	1.44 (0.92, 2.25)
≥3 meals/day	54 (9.9%)	489 (90.1%)	1	
Family size				
≤5	35 (7.8%)	411 (92.2%)	1	1
>5	64 (17.4%)	304 (82.6%)	2.47 (1.59, 3.83)	2.05 (1.31, 3.23)^*∗*^
Source of drinking water				
Improved	73 (10.2%)	642 (89.8%)	1	1
Nonimproved	26 (26.3%)	73 (73.7%)	3.13 (1.88, 5.21)	3.82 (2.20, 6.62)^*∗*^
Home latrine				
Yes	71 (10.7%)	590 (89.3%)	1	1
No	28 (18.3%)	125 (81.7%)	1.86 (1.15, 3.00)	0.97 (0.50, 1.85)
Mass media exposure				
High	74 (11.0%)	597 (89.0%)	1	
Low	25 (17.5%)	118 (82.5%)	1.70 (1.04, 2.80)	
Menstruation status				
Yes	57 (9.5%)	546 (90.5%)	1	1
No	42 (19.9%)	169 (80.1%)	2.38 (1.54, 3.67)	2.80 (1.75, 4.48)^*∗*^

*∗* = *p* value < 0.05. Signficant associated.
